# Yeast—As Bioremediator of Silver-Containing Synthetic Effluents

**DOI:** 10.3390/bioengineering10040398

**Published:** 2023-03-23

**Authors:** Inga Zinicovscaia, Nikita Yushin, Dmitrii Grozdov, Elena Rodlovskaya, Le Hong Khiem

**Affiliations:** 1 Joint Institute for Nuclear Research, Joliot-Curie Str., 6, 1419890 Dubna, Russia; 2 Horia Hulubei National Institute for R&D in Physics and Nuclear Engineering, 30, Reactorului str., 077125 Buharest, Romania; 3 A.N. Nesmeyanov Institute of Organoelement Compounds of Russian Academy of Sciences, Vavilova Str., 28, 119991 Moscow, Russia; 4 Institute of Physics of Vietnamese Academy of Science and Technology, Hanoi 700000, Vietnam

**Keywords:** waste biomass, *Saccharomyces cerevisiae*, silver-containing effluents, bioremediation, biosorption

## Abstract

Yeast *Saccharomyces cerevisiae* may be regarded as a cost-effective and environmentally friendly biosorbent for complex effluent treatment. The effect of pH, contact time, temperature, and silver concentration on metal removal from silver-containing synthetic effluents using *Saccharomyces cerevisiae* was examined. The biosorbent before and after biosorption process was analysed using Fourier-transform infrared spectroscopy, scanning electron microscopy, and neutron activation analysis. Maximum removal of silver ions, which constituted 94–99%, was attained at the pH 3.0, contact time 60 min, and temperature 20 °C. High removal of copper, zinc, and nickel ions (63–100%) was obtained at pH 3.0–6.0. The equilibrium results were described using Langmuir and Freundlich isotherm, while pseudo-first-order and pseudo-second-order models were applied to explain the kinetics of the biosorption. The Langmuir isotherm model and the pseudo-second-order model fitted better experimental data with maximum adsorption capacity in the range of 43.6–108 mg/g. The negative Gibbs energy values pointed at the feasibility and spontaneous character of the biosorption process. The possible mechanisms of metal ions removal were discussed. *Saccharomyces cerevisiae* have all necessary characteristics to be applied to the development of the technology of silver-containing effluents treatment.

## 1. Introduction

The development of high-technology products requires application of precious and strategic metals, including silver, gold, and rare earth elements [1]. The resource limitations and the possible environmental impacts evoked by metals, including silver, extraction are important prerequisites for their recovery from wastewater and wastes of electrical and electronic equipment [1]. Silver, due to its high electrical and thermal conductivity, antimicrobial properties, ductility, corrosion resistance, and malleability, is widely applied in medicine, the chemical industry, electroplating, jewelry production, photography, etc. [2,3,4,5,6,7]. The global silver demand in 2019 reached 30,848 t, while its production in the same year dropped by 1.3% [8]. According to Sverdrup and coauthors [9], by 2240 all silver mines will be nearly empty. 

However, it is equally important to prevent the release of silver ions into the environment, due to their high toxicity for living organisms [8]. Silver ion toxicity can be manifested by means of proteins, amino acids, and DNA damage, as well as impairment of the enzymes Na/K adenosine triphosphatase and carbonic anhydrase [10].

Currently, techniques such chemical precipitation, adsorption, ion exchange, membrane separation, ultrafiltration, reverse osmosis, and electrolysis are applied for silver ion recovery from wastewater [11,12,13]. Mentioned techniques allow recovery of 60 to 99% of silver ions from effluents [14,15]. However, most of these techniques, in addition to high cost and energy consumption, have a number of other shortcomings such as high reagent requirements and generation of toxic waste, which demand special treatment and storage conditions, and some of them are time-consuming [5,11,16].

Among applied techniques, adsorption, and in particular biosorption, is considered a promising technology due to its serious merits: low cost, availability of biosorbents, their easy recovery/reuse, generation of minimum amounts of sludge, high sorption capacity [11,16,17,18], and possibility of selective metal ion removal [19,20]. Biosorption is based on physico-chemical interactions between the metal ion and the functional groups present on the cell surface [10]. Many studies in the literature suggest the use of algae, bacteria, filamentous fungi, and yeast as biosorbents for metal ion removal from wastewater and batch systems [16,18,21,22,23,24,25,26,27,28,29].

*Saccharomyces cerevisiae* (*S. cerevisiae*) is a precious biosorbent with high metal removal capacity. The yeast is easy cultivated at large scale on inexpensive growth media [30,31]. *S. cerevisiae* is commonly used in the bakery and brewery industries [32,33] and results in generation of large quantity of waste yeast, which is easier to obtain [31,34,35]. In addition, the chemical composition of this yeast is well known, and it has high stability over time [21,36]. *S. cerevisiae* is a safe microorganism; therefore, it can be assumed that its application as biosorbent on a large scale will be easily accepted by the population [35]. Use of a flocculent strain of *S. cerevisiae* eliminates the need to immobilize the cells and results in higher ability to remove heavy metals from wastewater [37]. In Wang and Chen’s review [31] most of the works devoted to metal biosorption onto *S. cerevisiae* are found. It should be mentioned that mainly the process of metal uptake from mono solute systems was studied, whereas most commonly metals of interest in wastewater are accompanied by other chemical elements, and complex effluent treatment is required. The potential of *S. cerevisiae* to synthesize metal nanoparticles, including silver nanoparticles, has been reported [38,39]; however, to our knowledge there is no information about *S. cerevisiae* application for silver-containing effluent treatment. 

The aim of the present study was to (i) examine the effect of pH, silver concentration, contact time, and temperature on the sorption potential of *Saccharomyces cerevisiae* on metal removal from silver-bearing synthetic effluents with different chemical compositions; (ii) to explain kinetics of the process by applying pseudo-first-order and pseudo-second-order models, and equilibrium of the biosorption using Langmuir and Freundlich models; and (iii) to evaluate the thermodynamics of the process. The results obtained in the study can become the basis of the technology of *S. cerevisiae* use for silver-containing real effluent treatment.

## 2. Materials and Methods

### 2.1. Chemicals

Chemicals for experiments AgNO_3_, Cu(NO_3_)_2_·2.5H_2_O, HNO_3_, NaOH, and Ni(NO_3_)_2_·6H_2_O, Zn(NO_3_)_2_·6H_2_O of analytical grade were obtained from Sigma-Aldrich (Darmstadt, Germany).

### 2.2. Syntethic Effluents 

Since silver often is present in effluents along with copper, nickel, and zinc [40], three effluents with the following chemical compositions were prepared: Ag(I), Ag(I)/Cu(II), and Ag(I)/Cu(II)/Ni(II)/Zn(II). To prepare solutions with desired metal concentrations, a weighed quantity of salts was dissolved in bidistillated water (1 L for each solution) and obtained solutions were immediately used for experiments. The concentration of metal ions in all systems was: 10 mg/L for silver (15.7 mg of salt), 5 mg/L for copper (18.3 mg of salt), and 2 mg/L for zinc and nickel (9.1 mg and 9.9 mg of salts, respectively). 

### 2.3. Biosorbent 

Yeast *Saccharomyces cerevisiae* biomass used as biosorbent is a waste product generated by a brewing company (Efes Vitanta Moldova Brewery, Chisinau, Republic of Moldova). Before application in biosorption experiments, biomass was dried at 105 °C and homogenized. 

### 2.4. Metal Removal from Silver-Containing Effluents

Single-metal biosorption experiments were performed in a conic flask of 100 mL volume using 50 mL of solution containing silver in a concentration of 10 mg/L and 500 mg of biosorbent. The effect of pH on silver ion removal was assessed in the pH range 2.0–6.0. The pH of the experimental solutions was adjusted to the required values of HNO_3_ or NaOH solutions. To obtain equilibrium, isotherm silver ion concentrations in solution varied from 10 to 100 mg/L at fixing pH (3.0), contact time (60 min), and temperature (22 °C). For kinetics, samples were withdrawn at predetermined time intervals (3, 7, 15, 30, 45, 60, and 120 min), maintaining pH (3.0), silver concentration (10 mg/L), and temperature (22 °C). The thermodynamics of the process was assessed by varying the temperature of the solution from 20 to 50 °C, keeping pH (3.0), silver concentration (10 mg/L), and time (60 min) constant. 

In multicomponent systems, the effect of the same parameters on metal removal by yeast biomass was assessed. The effect of pH on metal ion removal was studied in the pH range 2.0–6.0. To obtain experimental data for estimation of equilibrium, kinetics, and thermodynamics of the biosorption, the experiments were carried out according to the scheme described for single-metal biosorption, varying time (3–120 min), silver concentration (10–100 mg/L), or temperature (20–50 °C), and other parameters were maintained as invariable. It should be mentioned that copper, nickel, and zinc concentrations in solutions in performed experiments were held constant. 

Upon completion of the experiments, biosorbent separated from the solution was dried at 105 °C and used for the determination of silver, nickel, and zinc uptake, whereas experimental solutions were used to determine copper concentration. During biosorption experiments, precipitate formation or change of the experimental solutions’ color were not seen. 

### 2.5. Metal Desorption

To assess metal ions desorption, yeast biomass firstly came in contact with silver-containing synthetic effluents at optimum experimental conditions. Next, the biomass was separated from the solution by centrifugation. The obtained metal-loaded biosorbent was then brought in contact with 50 mL of 0.1 M HNO_3_ and EDTA for 60 min in an orbital shaker at 200 rpm. Each desorption experiment was performed in triplicate. The desorption rate was calculated using measurements of metal concentration in solutions. 

### 2.6. Methods

The sorption of silver, nickel, and zinc on *S. cerevisiae*, as well as the content of calcium, sodium, and potassium in biomass, were determined using neutron activation analysis at the IBR-2 reactor (Joint Institute for Nuclear Research, Dubna, Russia). The content of calcium was determined after sample irradiation for 3 min and measurement for 15 min. The content of other elements was determined after sample irradiation for 72 h at a neutron flux of 1.2 × 10^12^ cm^−2^ s^−1^ and measurement after 4 and 20 days of irradiation for 30 min (sodium and potassium) and 90 min (zinc, nickel and silver), respectively, using HP-Ge detectors. Gamma spectra processing and metal content determination were performed using software Genie 2000 and Concentration. Copper concentration in solutions was determined by applying an atomic absorption spectrometer (iCE 3400, Thermo Fisher Scientific, Waltham, MA, USA). Infrared spectra were obtained by applying the Bruker Alpha Platinum-ATR spectrometer (Bruker Optics, Ettingen, Germany), while morphology of the cells was analyzed using an S3400N Hitachi microscope (Krefeld, Germany).

The amount of metal ions adsorbed per gram of biosorbent (q, mg/g) and efficiency of the removal process (%) were defined by the Equations (1) and (2):

(1) $$q=\frac{V\left(C_{i}-C_{f}\right)}{m}$$

(2) $$R=\frac{C_{i}-C_{f}}{C_{i}}\times 100$$

where q is adsorption capacity, mg/g; V is the volume of solution, L; C_i_ and C_f_ metal concentrations at the beginning g and end of the biosorption process, mg/L; and m is the mass of yeast, g.

## 3. Results and Discussion

### 3.1. Biosorbent Characterization

**Figure 1:**
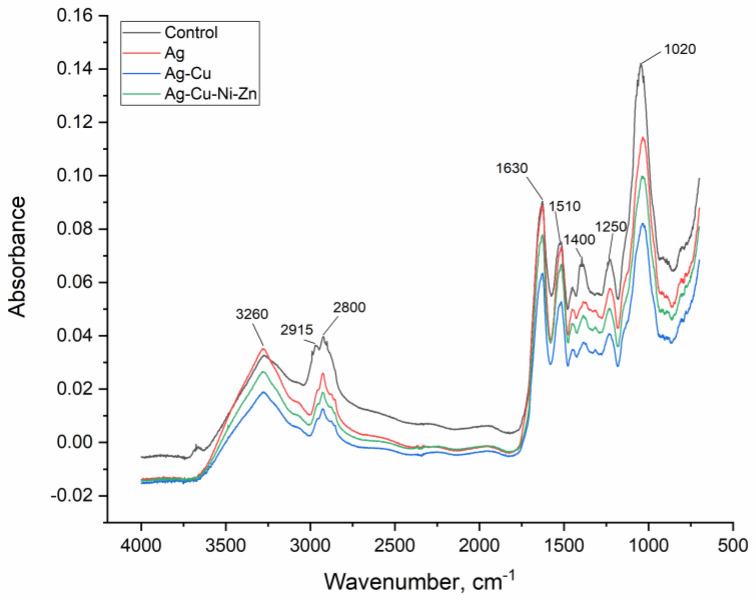
FTIR spectra of *S. cerevisiae* biomass before and after silver-containing effluent treatment.

Fourier-transform infrared spectroscopy (FTIR) and Scanning Electron Microscopy (SEM) were applied for biosorbent characterization. To determine the contribution of functional groups to metal binding, *S. cerevisiae* raw biomass and biomass after treatment of silver-containing effluents were subjected to FTIR analysis (Figure 1). FTIR spectrum of native yeast biomass showed several intensive adsorption bands in the region of 1020 and 1510 cm^−1^, which correspond to the vibrations of OH- groups and amide I–III bands of polypeptide/proteins. In the region, 1225 cm^−1^ were present stretching vibrations of the C=O group, while bands of and 2800 cm^−1^ appertain to vibrations of alkyl groups, CH, -CH_3_, or -CH_2_. The peak at 1630 cm^−1^ may be related to CH=CH groups from alkenes as well as =O (carbonyl) and N-H (amide) deformation bands [41]. The adsorption band at 3260 cm^−1^ could be attributed to hydroxyl (–OH) and amine (–NH) functional groups [34,42]. The peak at 2915 cm^−1^ is characteristic for methylene C-H stretching vibrations [41]. The peaks at the 1238 and 1045 cm^−1^ regions are attributed to the presence of a sulfo group and P=O stretching vibration [34,41]. 

In the spectra of metal-loaded biomass, there has been no formation of new bands, indicating that the biosorption of studied metal ions process occurs mainly through ion-exchange interaction that is electrostatic [21,43]. Thus, in the spectrum of yeast biomass after silver ion biosorption, the shift of the bands corresponding to OH, CH=CH, –NH, and sulfo groups by 5–15 cm^−1^ was observed indicating their involvement in silver ion binding. According to [34], hydroxyl and sulfo groups play a main role in silver ion biosorption. 

In the Ag(I)/Cu(II) spectrum, the position of OH, CH=CH, CH_2_/CH_3_, NH_2_, and sulfo groups was shifted by 7–15 cm^−1^, pointing at their participation in metal binding. It should be mentioned that Ag-system alkyl groups did not participate in silver ion binding; thus, it can be concluded that copper ions are trapped by these groups. It was previously reported that OH, CH=CH, C=O, and N=O groups participate in copper ion binding [44]. Brady and Duncan [45] reported main role of amino, carboxyl, or hydroxyl groups in copper ion removal. In the Ag(I)/Cu(II)/Ni)II)/Zn(II) system, all functional groups defined in *S. cerevisiae* biomass were involved in metal ion binding. Carboxyl, amino, hydroxyl, and amide groups of protein and carbohydrate participated in copper, zinc, and nickel uptake by *S. cerevisiae* [24]. Savastru et al. [21] found that removal of Zn(II) and Cu(II) by *S. cerevisiae* occurred predominantly through ion exchange interactions, with the hydroxyl and carboxyl groups playing the metal role in binding process. 

**Figure 2:**
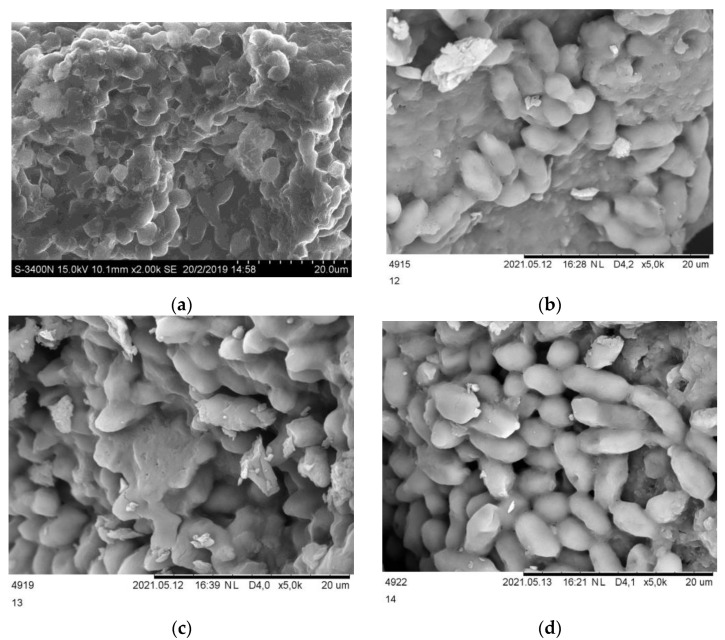
SEM images of *S. cerevisiae* biomass (**a**) control, (**b**) Ag(I) system, (**c**) Ag(I)/Cu)(II) system, and (**d**) Ag(I)/Cu(II)/Ni(II)/Zn(II) system.

The SEM microphotographs of *S. cerevisiae* before and after metal uptake are shown in Figure 2. 

Metal adsorption onto yeast is considered a typical passive biosorption process [46]. Before metal biosorption, yeast cells had rough cell surface, being overlapped with each other. The SEM micrographs of biomass after silver-containing effluent treatment did not show remarkable changes in cell structure in comparison with control. The rough surface of the yeast cells allows easier access for metal ions to reach the active sites, which facilitates the biosorption process [21]. Therefore, it can be suggested that metal biosorption occurs on the cell surface [41]. No aggregates of metal complex deposited in the form of granules on the cell surface were observed; thus, it can be concluded that sorption takes place through surface complexation, coordination, or ion exchange [47]. 

### 3.2. Effect of pH on Metal Removal from Silver Containing Effluents

**Figure 3:**
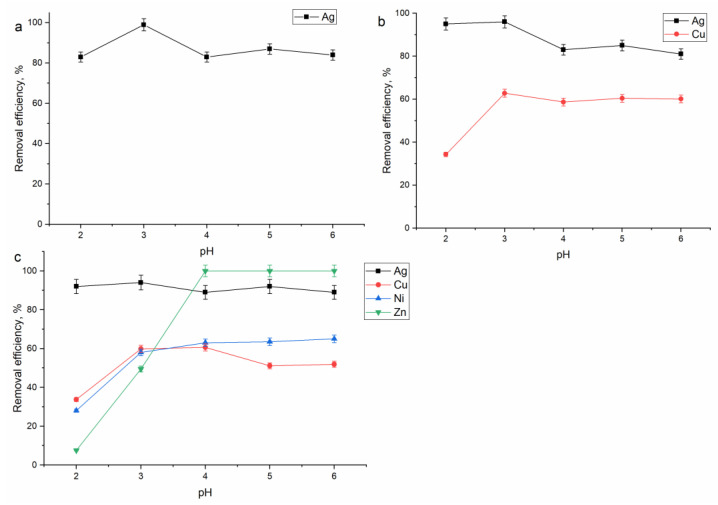
Effect of solution acidity on metal ion removal from batch solutions by *S. cerevisiae* biomass: (**a**) Ag(I) system, (**b**) Ag(I)/Cu(II) system, and (**c**) Ag(I)/Cu(II)/Ni(II)/Zn(II) system (time: 1 h, temperature: 20 °C, pH: 2.0–6.0, C_i,Ag_: 10 mg/L, C_i,Cu_: 5 mg/L, C_i,Ni, Zn_: 2 mg/L).

The pH of the solution is an important parameter that can ensure high metal ion removal due to its effect on metal ion chemistry and charge of the functional groups [11]. Experiments were performed at pH range of 2.0–6.0, when silver is present in solution in form of Ag(I), and while a pH higher than 6.0 AgOH is generated [16,34]. In all analyzed systems, maximum removal of silver ions was attained at pH 3.0, when 94–99% of metal ions were removed from solutions (Figure 3). It should be mentioned that for the rest of the pH values, the removal of silver ions by *S. cerevisiae* was at the quite high level of 81–92%. Thus, it can be concluded that *S. cerevisiae* can be applied for silver-containing effluent treatment at the studied pH range. 

Generally, at low pH values, competition of metal ions with H^+^ ions in the solution may occur [48]. At the same time, relatively high efficiency of silver ion removal at pH 2.0 can be explained by their binding to amino groups [49]. With the pH increase, the number of available negatively charged groups increases, thereby facilitating uptake of elements present in the solution in cationic form. A slight decrease of the silver removal at the pH range 4.0–6.0 can be explained as the use of sodium hydroxide to raise the pH of the solutions [50]. 

In the Ag(I)/Cu(II) system, copper removal increased from 38% at pH 2.0 to 63% at pH 3.0, and it was maintained at almost the same level. In [50], copper sorption onto *S. cerevisiae* was studied at pH 6.0. Maximum copper uptake by *S. cerevisiae* from a multimetal system was achieved at pH 3.0 [44]. The efficiency of silver removal in Ag(I) and Ag(I)/Cu(II) systems was almost on the same level, indicating that silver and copper ions bind to different functional groups. 

Furthermore, it is interesting to mention that silver ion removal at pH 2.0 increases in systems containing copper ions. It can be suggested that copper ions could serve as new sites for silver ion removal by forming surface ternary complexes ([-N -Cu]–Ag) [51].

In the Ag(I)/Cu(II)/Ni(II)/Zn(II) system, increase of solution pH from 2.0 to 6.0 resulted in the increase of nickel ion removal increasing from 28 to 65%. Maximum removal of zinc (100%) and copper (61%) was attained at pH 3.0. Zinc removal was maintained at the level of 100% at the pH range of 4.0–6.0, while the level of silver and copper ion removal was comparable with previously described systems. The high degree of nickel and zinc removal at pH 5.0–6.0 is explained by their precipitation at high pH value: 8.4 for zinc and 8.7 for nickel, while copper precipitates at pH 6.4 [44]. 

Maximum removal of zinc from complex effluents by *S. cerevisiae* was attained at pH range 3.0–6.0 and of nickel at pH 6.0 [52,53]. Since silver ions were of main interest, further experiments were performed at pH 3.0. 

### 3.3. Effect of Time on Metal Biosorption on S. cerevisiae Biomass and Kinetic Studies

**Figure 4:**
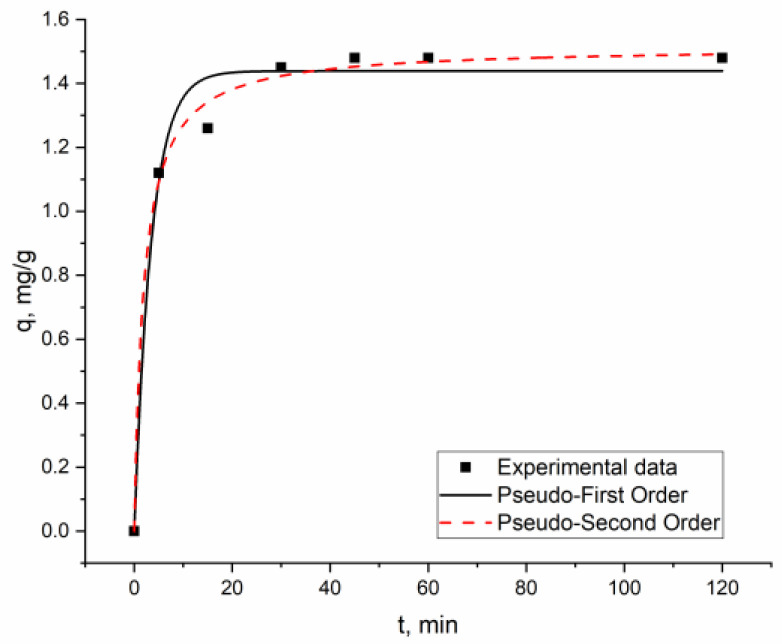
The adsorption kinetic of silver ion biosorption on yeast biomass in Ag(I) system.

**Figure 5:**
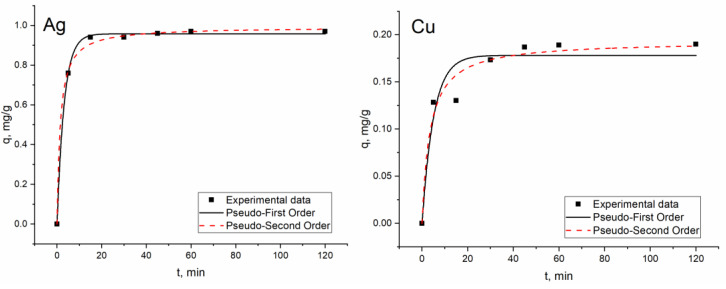
The adsorption kinetic of silver and copper ion biosorption on yeast biomass in Ag(I)/Cu(II) system.

**Figure 6:**
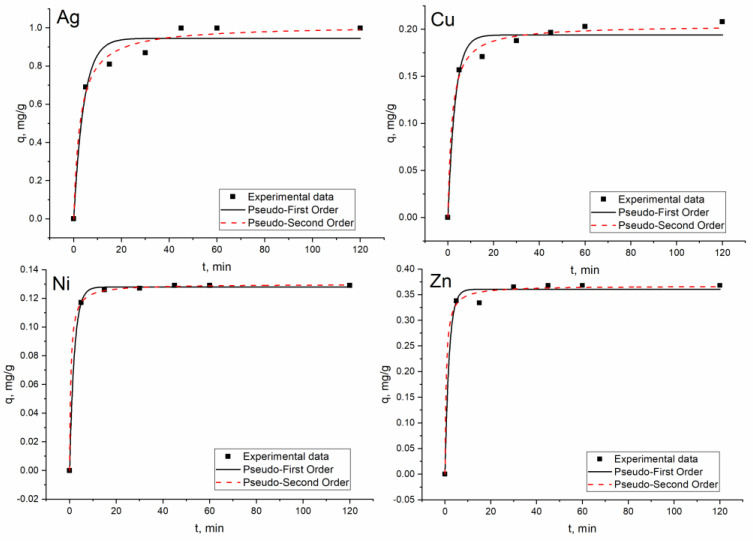
The adsorption kinetic of metal ion biosorption on yeast biomass in Ag(I)/Cu(II)/Ni(II)/Zn(II) system.

The effect of time on the biosorption of metal ions present in the analyzed system is shown in Figure 4, Figure 5 and Figure 6. At the adsorption time 5–45 min, yeast biomass sorption capacity for silver ions increased sharply; then the rate was lowered, and equilibrium was attained. At the same time, it should be mentioned that the most remarkable increase of silver ion adsorption took place in the first 5 min of sorbent interaction with sorbate. The efficiency of silver ion removal varied from 97% in case of the Ag(I)/Cu(II) system to 100% in the Ag(I)/Cu(II)/Ni(II)/Zn(II) system. The removal of other metal ions was also a two-stage process characterized by rapid metal ion uptake in the first stage and equilibrium attainment in the second stage. Thus, 57–65% of copper ions (both systems), 99% of nickel ions, and 84% of zinc ions were removed from the analyzed systems during a 2 h experiment. High adsorption of metal ions on yeast biomass can be explained by the high values of the affinity constants between metal ions and yeast biomass [53]. It should be mentioned that affinity constants for anions that are present in the solution are considerably lower compared with cations; thus, metal uptake from solutions would not be influenced by the presence of anionic ligands [53]. High removal of metal ions can also be associated with the ability of *S. cerevisiae* to flocculate in the presence of zinc, copper, and nickel ions, thus increasing its sorption capacity [37]. Thus, yeast biomass preference for the metal ions can be described as follows: Ag > Ni > Zn > Cu.

At the initial stage of the sorbent interaction with sorbate, the number of unoccupied functional groups on the *S. cerevisiae* surface is relatively high, facilitating rapid binding of the metal ions. Increasing the interaction time results in the decrease of the number of unoccupied binding sites, thereby lowering the biosorbent removal capacity [21,42]. The quick time required for equilibrium achievement (45–60 min) suggested that metal ion biosorption occurs mainly on the surface of yeast via electrostatic interactions [21].

Rapid adsorption of metal ions onto *S. cerevisiae* is in agreement with previously performed studies [34,54,55]. The adsorption of silver ions on immobilized coffee grounds was almost completed within 60 min [17]. 

The experimental data were described using pseudo-first-order and pseudo-second-order models.

The pseudo-first-order model is used to describe a one-site-occupancy adsorption when the adsorbing molecule reacts with one adsorption site [56]:

(3) $$q_{t}={\;q}_{e\;}(1-e^{-k_{1}t})$$

where q_e_ and q_t_ are the amount of metal ions (mg/g) adsorbed from the analyzed systems at equilibrium and t time, min; and k_1_ is the rate constant of the pseudo-first-order model, 1/min. 

The pseudo-second-order model is based on the assumption that chemical sorption is the rate-limiting step of the sorption process:

(4) $$q=\frac{q_{e}^{2}k_{2}t}{1+q_{e}k_{2}t}$$

where k_2_ is the rate constant of the pseudo-second-order model, g/mg·min.

The applicability of kinetic models was confirmed by SSE (sum of error squares), %:

(5) $$\mathrm{SSE}=\frac{\sqrt{\sum \;{\left(q_{e,\;\mathrm{cal}}-q_{e,\;\exp}\right)}^{2}}}{N}$$

where N is the number of experimental points.

The nonlinear fitting of the experimental data is presented in Figure 4, Figure 5 and Figure 6, and fitting parameters are displayed in Table 1. 

According to coefficients of determination values and close adsorption capacity values calculated and obtained experimentally, it can be concluded that both models can be applicable for the explanation of the experimental data. However, the results of the Akaike Information Criterion (AIC) test showed that biosorption of metal ions present in analyzed systems fitted better with the pseudo-second-order model. This is in agreement with Staron et al. [10] and Zhao et al. [34], who used coconut fiber and graphene oxide for silver sorption. Applicability of the pseudo-second model suggests that metal ion binding on the surface of *S. cerevisiae* is achieved via chemical interactions that include two active centers in favorable geometric positions [43]. It is suggested that the adsorption of the investigated metal ions occurs mainly due to ion exchange and surface complexation reactions at specific biosorption sites [34,57]. The results of neutron activation analysis confirmed the importance of ion-exchange process in studied metal ion removal. In particular, the content of Na, Ca, and K in metal-loaded biomass was reduced in comparison with control biomass 2.0–5.0 times, 1.2–1.7 times, and 1.4–2.6 times, respectively. During the biosorption of silver by a waste product from the alginate production, the release of light metal cations, especially sodium and calcium, was observed [49].

**Figure 7:**
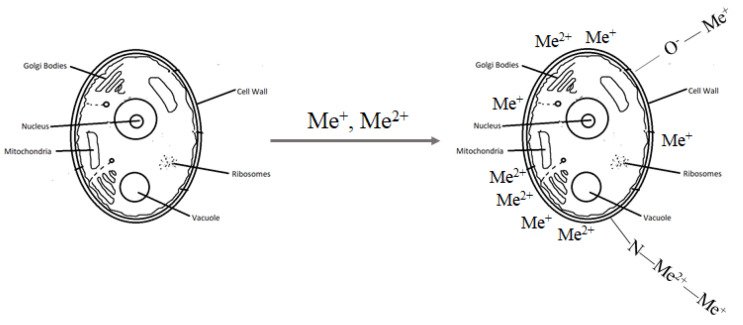
A skeletal representation of metal ion biosorption on yeast cell.

The schematic representation of the metal ion biosorption on *S. cerevisiae* is presented in Figure 7. 

### 3.4. Effect of Silver Concentration on Metal Ion Biosorption on S. cerevisiae Biomass and Equlibrium Studies

**Figure 8:**
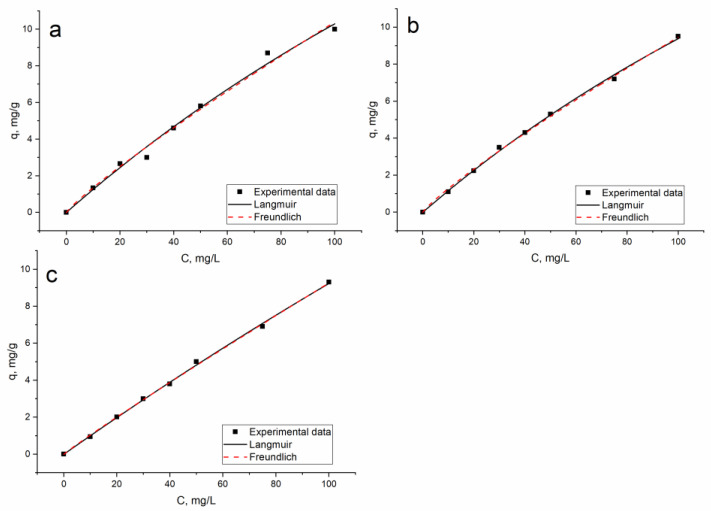
The sorption isotherms for silver ion biosorption on the *S. cerevisiae* biomass: (**a**) Ag(I) system, (**b**) Ag(I)/Cu(II) system, and (**c**) Ag(I)/Cu(II)/Ni(II)/Zn(II) system.

In biosorption experiments, the concentration of metal ions can significantly affect sorbents’ biosorption capacity. Increasing of the silver ion concentration in experimental solutions from 10 to 100 mg/L resulted in the increase of *S. cerevisiae* biomass sorption capacity from 1.3 to 10 mg/g in the Ag(I) system, from 1.1 to 9.5 mg/g in the Ag(I)/Cu(II) system, and from 0.94 to 9.3 mg/g in the Ag(I)/Cu(II)/Ni(II)/Zn(II) system (Figure 8). At low metal ion concentrations in solution, their high sorption is explained by the large number of active sites on the surface of the adsorbent, while at high metal concentrations the efficiency of sorption decrease is due to occupation of binding sites [25]. However, as it can be seen from the experimental data presented in Figure 8, *S. cerevisiae* biomass maintained high sorption capacity toward silver ions even at high metal ion concentrations in solution. Thus, at silver concentrations in solution below 100 mg/L, *S. cerevisiae* biomass was able to almost completely remove silver ions. This is in line with results reported by Zhao et al. [34], who studied silver removal from single metal systems and showed that yeast biomass can be efficiently applied for single- and multi-metal silver-containing effluent treatment. According to Chowdhury et al. [58], a rise of the initial metal concentration in solution provides a driving force to overcome all mass transfer resistances of metal ions between the aqueous and solid phase, facilitating metal uptake.

The presence of copper, zinc, and nickel ions in the analyzed systems did not significantly influence adsorption of silver ions in comparison with the Ag(I) system. At the same time, it should be noted that copper (in both systems) and nickel ion removal was practically unaffected by the change of the silver ion concentration. Zinc was the only element for which removal decreased from 88% at silver concentration in solution of 10 mg/L to 40% at silver concentration of 100 mg/L. In previously performed studies, it was shown that in complex systems where zinc, coper, and nickel ions are present their sorption did not differ considerably from singe metal systems [59]. Decrease of zinc removal can be explained for its competition with silver ions for binding sites with the increase of silver concentration in solution. In complex effluents, metal ions compete for binding sites; as a consequence, displacement occurs of one metal species by another, which has higher affinity for biomass binding sites [53]. 

To describe silver behavior during the biosorption process, the Langmuir and Freundlich isotherms were applied. The Langmuir model describes a monolayer and uniform sorption [17]:

(6) $$q_{e}=\frac{q_{m\;}{\mathrm{bC}}_{e}}{1+{\mathrm{bC}}_{e}}$$

where q_m_ is the maximum adsorption capacity of the yeast biomass, mg/g; and b is the adsorption equilibrium constant, L/mg.

The Freundlich model assumes as a hypothesis an infinite number of adsorption sites and is applicable for the description of sorption on heterogeneous surfaces [17]:

(7) $$q_{e}=K_{F}{\mathrm{Ce}}^{\frac{1}{n}}$$

where K_F_, L/mg, and n are the constants of adsorption affinity.

Separation factor R_L_, which predicts the potential adsorption probability relationship between solid and liquid, was calculated using Equation (8):

(8) $$R_{L}=\frac{1}{1+{\mathrm{bC}}_{0}}$$

where 0 < R_L_ < 1 indicates favorable adsorption, R_L_ > 1—unfavorable adsorption, R_L_ = 1—linear adsorption, and R_L_ = 0—irreversible adsorption [52].

The nonlinear fitting of the experimental data is illustrated in Figure 8, while isotherm parameters are summarized in Table 2. High values of the coefficient of correlation were obtained for both applied models. Since both models showed good results, the AIC test was applied to find the most appropriate model for the metal silver ion adsorption. According to AIC test results, experimental data for all analyzed systems were better fitted to the Langmuir isotherm model. The Langmuir isotherm model assumes that the surface of the biosorbent is homogenous in nature, and all the binding sites are uniformly distributed, having the same affinity. Once a site is occupied, no further sorption can take place at that site [28,60]. The maximum adsorption capacity calculated from the Langmuir model ranged from 43.6 mg/g in the Ag system to 108 mg/g in the Ag/Cu/Ni/Zn system and was considerably higher compared to the experimentally obtained values, suggesting that a much larger number of metal ions is needed to form monolayer coverage [21]. The Freundlich constant n ranged between 1.11 and 1.15, indicating that adsorption is a physical process [28]. The R_L_ values between 0 and 1 indicate favorable biosorption of silver ions on *S. cerevisiae* biomass. Since the coefficients of correlation for the Freundlich model, which assumes heterogeneous adsorption due to the diversity of the adsorption sites [34], were also high, it can be suggested that silver biosorption is a complex process, in which specific functional groups play a dominant role in silver ion binding.

The sorption of silver ions on coconut fiber was described using Langmuir and Freundlich models [10]. The biosorption of silver by a waste product from the alginate production industry was also better described by the Freundlich model [49].

The comparison of the sorption capacity of a series of alternative sorbents is given in Table 3. The values obtained in the present study were comparable with the data reported in the literature. Here, it is important to mention that experiments were performed at different optimal experimental conditions. 

### 3.5. Effect of Temperature on Metal Ion Biosorption on on S. cerevisiae Biomass and Thermodinamic Studies

**Figure 9:**
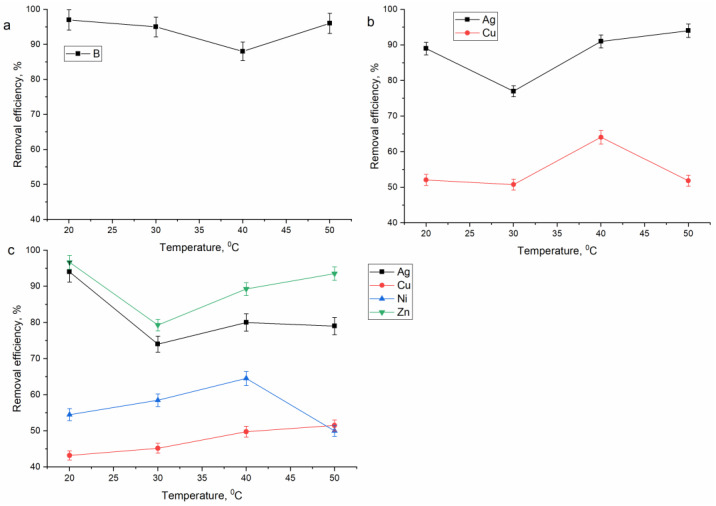
Effect of temperature on metal ion removal by *S. cerevisiae* (**a**) Ag(I) system, (**b**) Ag(I)/Cu(II) system, and (**c**) Ag(I)/Cu(II)/Ni(II)/Zn(II) system (time 1 h, pH 3.0, C_i,Ag_ 10 mg/L, C_i,Cu_ 5 mg/L, C_i,Ni, Zn_ 2 mg/L).

Temperature of the solution can significantly affect metal removal. The behavior of silver ion removal as a function of the temperature of a solution differed between studied systems (Figure 9). In the Ag(I) system, at temperatures 20 and 30 °C, silver ion removal was almost on the same level (95–96%), and it slightly decreased (less than 10%) at temperature 40 °C. Further increase of the temperature up to 50 °C has returned removal efficiency at the level of 96%. In the Ag(I)/Cu(II) system, silver ion removal firstly dropped by approximately 10% with a temperature increase to 30 °C; then it rose to 94% at 50 °C. For copper, maximum removal of 64% was attained at 40 °C, while at the other temperatures, it was at the level of 52%. In the Ag(I)/Cu(II)/Ni(II)/Zn(II) system, the removal of silver ions decreased by 20% (from 94 to 74%) with a temperature increase from 20 to 30 °C, and it did not change with further temperature growth. Maximum removal of copper (52%) and of nickel (64.5%) was attained at 50 °C and 40 °C, respectively. The behavior of zinc ion removal was similar to those observed for silver ions in the Ag(I) system. The decrease of metal ion removal at temperatures between 30 and 40 °C can be explained by the weakening of the structure of the biomass cell and possible degradation of the biomolecules resulting in a decrease of active binding sites [67]. The increase of metal ions with the rise in the temperature can be associated with the reduction of the solution viscosity and an increase in the mobility of the metal ions in the solution [10].

The thermodynamic study was undertaken to calculate free energy ΔG°, ΔH° (J/mol), and ΔS° (J/mol) values (9)–(11):

(9) $${\mathrm{lnK}}_{d}=\frac{{\Delta S}^{{}^{\circ}}}{R}-\frac{{\Delta H}^{{}^{\circ}}}{\mathrm{RT}}$$

(10) $$\Delta G^{{}^{\circ}}=\Delta H^{{}^{\circ}}+T\Delta S^{{}^{\circ}}$$

where K_d_ is the distribution coefficient, and it is calculated according to Equation (10):

(11) $$K_{d}=\frac{\left(C_{0}-C_{e}\right)V}{{\mathrm{mC}}_{e}}$$

where ΔH° and ΔS° are enthalpy and entropy changes, respectively; R is the universal gas constant; 8.314 J/mol K; and T is the absolute temperature, K. 

The enthalpy and entropy of the process were determined from the slopes and intercept of the plot of ln*K_d_* versus 1/*T* (Supplementary Figure S1), and the obtained thermodynamic parameters are presented in Table 4. The negative values of the ΔG° obtained for all elements confirm a spontaneous nature of the metal ion biosorption on the *S. cerevisiae* biomass. It is considered that Gibbs free energy in the range of 0 to −20 kJ/mol indicates physical adsorption, −20 to −80 kJ/mol ion exchange, and −80 to −400 kJ/mol chemisorption [68]. According to data obtained in the present study, physical adsorption can be suggested as main mechanism of metal ion removal. 

The positive values of enthalpy for elements in the Ag(I)/Cu(II) system and copper and nickel ions in the Ag(I)/Cu(II)/Ni(II)/Zn(II) system suggested an endothermic character of their biosorption, which is unequivocally attributable to chemisorption [69]. An exothermic process signifies either physical sorption or chemisorption, while endothermic process is mainly associated with chemisorption [70]. For other elements, negative values of ΔH° points at an exothermic character of biosorption. The positive value of ΔS° obtained for all elements, except silver ions in the Ag(I)/Cu(II)/Ni(II)/Zn(II) system, indicated an increment in the randomness in the system solid/solution interface during the biosorption [71,72]. This suggests that metal ions present in analyzed system can replace some water molecules in the solution earlier adsorbed on the surface of the adsorbent [69]. 

**Figure 10:**
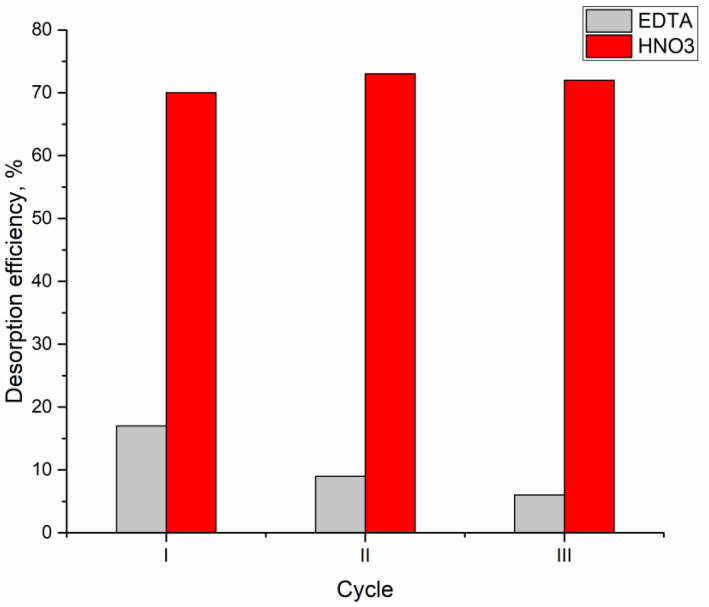
The efficiency of silver ion desorption using as eluents EDTA and HNO_3_.

During three sorption–desorption cycles, the efficiency of silver ion desorption in studied systems was on the level of 17% when EDTA was used as eluent and up to 73% when HNO_3_ was applied (Figure 10). 

During biosorbent regeneration, the adsorbent and eluent interact via electrostatic reactions, π– π bond reaction, hydrophobic reactions, and hydrogen bond reactions [73]. HNO_3_ is a proton exchange agent, which has been shown to be very efficient for metal cations desorption [73,74]. 

HNO_3_ was more suitable for copper, zinc, and nickel ion desorption, and the desorption efficiency was on the level of 60–70%. This is in agreement with previously performed studies [75]. In the case of EDTA desorption, efficiency did not exceed 10% (Figures S2 and S3). As it was reported in [76], the desorbing effect of EDTA is the highest at pH range of 3.0–5.0. Low metal desorption in present study can be explained with the increase of the pH of experimental mixture when adding EDTA to biomass up to 6.5. It should be mentioned that yeast biomass maintained high biosorption capacity during three sorption–desorption cycles. 

## 4. Conclusions

*S. cerevisiae* biomass was applied to study metal ion removal from synthetic silver-containing effluents under different pH, silver concentration, contact time, and temperature. The process of metal removal was quick and pH-dependent. Maximum removal of silver and copper ions in all systems was achieved at pH 3.0, of nickel ions at pH 6.0, and of zinc ions at pH range 3.0–6.0. Metal ion biosorption was a two-phase process; the first phase was characterized by quick metal ion removal, and the second stage was characterized by equilibrium attainment after 45–60 min of sorbent–sorbate interaction. The Langmuir isotherm model best fitted the experimental data, and maximum sorption capacity of the *S. cerevisiae* biomass varied from 43.6 to 108 mg/g. At silver concentration in solution in the range of 10–100 mg/L, it was possible to remove 93–100% of silver; that indicated a high sorption capacity of yeast biomass. The kinetics of the process were better represented by a pseudo-second-order model, indicating that chemisorption plays a main role in metal ion removal. Based on the obtained data, it can be concluded that binding of metal ions on yeast biomass may occur through electrostatic interaction, displacement of metal cation, or protons and complexation. According to thermodynamic parameters, biosorption was a favorable spontaneous process with exothermic or endothermic character depending on metal ions. Yeast biomass maintained high adsorption capacity during three sorption–desorption cycles, and HNO_3_ showed to be more suitable for metal ion desorption. 

The high biosorption capacity of *S. cerevisiae* biomass allows production of an eco-friendly and cheap biosorbent that is easy to handle and can be applied to treatment of large volumes of wastewater. As limitations of the proposed technology can be mentioned, its applicability is limited at the pH range lower that than those at which metal ions form precipitates and further utilization of the spent sorbent.

## Figures and Tables

**Table 1:** The fitting results of the parameters calculated from pseudo-first-order (PFO) and pseudo-second-order (PSO) models.

		Ag	Ag/Cu		Ag/Cu/Ni/Zn			
	Metal	Ag	Ag	Cu	Ag	Cu	Ni	Zn
	*q_exp_*	1.48 ± 0.02	0.97 ± 0.004	0.19 ± 0.03	0.99 ± 0.01	0.20 ± 0.003	0.13 ± 0.004	0.37 ± 0.003
PFO	*q_e_*	1.44 ± 0.03	0.96 ± 0.005	0.18 ± 0.09	0.96 ± 0.03	0.19 ± 0.005	0.12 ± 0.005	0.26 ± 0.005
	*k_1_*	0.28 ± 0.04	0.3 ± 0.01	0.2 ± 0.06	0.23 ± 0.05	0.3 ± 0.06	0.40 ± 0.02	0.55 ± 0.01
	*R^2^*	0.98	0.99	0.91	0.96	0.97	0.99	0.99
	SSE	0.09	0.08	0.19	0.07	0.02	0.91	0.18
PSO	*q_e_*	1.51 ± 0.02	0.99 ± 0.01	0.19 ± 0.009	1.01 ± 0.01	0.20 ± 0.004	0.13 ± 0.002	0.37 ± 0.005
	*k_2_*	0.3 ± 0.06	0.7 ± 0.09	1.5 ± 0.05	0.4 ± 0.09	2.7 ± 0.6	1.4 ± 0.7	5.1 ± 0.5
	*R^2^*	0.99	0.99	0.96	0.98	0.99	0.99	0.99
	SSE	0.17	0.16	0.31	0.11	0.02	1.1	0.22

**Table 2:** The parameters of the Langmuir and Freundlich models applied to describe silver biosorption.

		Ag	Ag/Cu	Ag/Cu/Ni/Zn
	*q_m_*, mg/g	51 ± 2.1	43.6 ± 2.5	108 ± 4
Langmuir	*b*, L/mg	0.002 ± 0.0001	0.003 ± 0.0002	0.0009 ± 0.0002
	*R_L_*	0.8	0.8	09
	*R* ^2^	0.99	0.99	0.99
	*R_adj_^2^*	0.98	0.98	0.98
Freundlich	*K_F_*, mg/g	0.18 ± 0.004	0.17 ± 0.06	0.11 ± 0.001
	*n*	1.13 ± 0.07	1.15 ± 0.02	1.11 ± 0.02
	*R* ^2^	0.99	0.99	0.99
	*R*_adj_^2^	0.98	0.98	0.98

**Table 3:** Adsorptive capacity of different types of sorbents used for silver ion sorption.

Sorbent	*q_max_*, mg/g	Concentrations Range, mg/L	pH	Reference
*S. cerevisiae* biomass	43.6	10–100	3.0	Present study
Immobilized coffee ground beads	39.583	10–300	6.0	[17]
Graphene oxide in different modifications	11.3–123	0–150	5.0	[8]
Waste yeast	18.9–41.8	0–750	3.0	[34]
Coconut fiber	65–82	5–30	3.0	[10]
Poly(o-phenylenediamine) microparticles	533	1–10 mM	5.0	[61]
*Spirulina platensis*	31.6	5–30	5.0	[55]
Chitosan/montmorillonite	43.48	1–10	6.0–7.0	[7]
Aniline–sulfoanisidine copolymer nanosorbent	2034	200–400	6.0	[62]
Wine industry wastes (grape peel, seed, and stem)	41.7–61.4	25–300	7.0	[63]
Exopolysaccharide produced by marine bacteria	256	-		[64]
*Salvinia Cucullata*	21.1	2–500	6.0	[65]
*Paecilomyces Lilacinus*	1010	-	3.0	[66]

**Table 4:** The estimated thermodynamic parameters for metal ion biosorption on *S. cerevisiae* biomass.

System	Metal	∆*G*^◦^, kJ/mol				∆*H*°, kJ/mol	∆*S*°, J/mol·K
		293 K	303 K	313 K	323 K		
Ag	Ag	−19.0	−19.1	−19.2	−19.4	−15.3	12.3
Ag/Cu	Ag	−15.1	−16.5	−17.8	−19.1	23.5	132
	Cu	−9.9	−10.3	−10.7	−11.1	1.6	39.0
Ag/Cu/Ni/Zn	Ag	−16.7	−16.2	−15.7	−15.2	−31.8	−51.5
	Cu	−11.2	−11.9	−12.6	−13.2	8.2	66.5
	Ni	−18.1	−18.7	−19.4	−20.1	1.1	65.6
	Zn	−15.4	−15.6	−15.9	−16.1	−8.3	24.1

## Data Availability

All data are presented in the manuscript.

## Supplementary Materials

The following supporting information can be downloaded at: https://www.mdpi.com/article/10.3390/bioengineering10040398/s1, Figure S1: Plot of lnK_d_ versus 1/T, Figure S2: The efficiency of metal ions desorption using as eluents EDTA and HNO_3_ in the Ag/Cu system, Figure S3: The efficiency of metal ion desorption using as eluents EDTA and HNO_3_ in the Ag/Cu/Ni/Zn system.
