# Supplementary material for: Yeast—As Bioremediator of Silver-Containing Synthetic Effluents
Source: Bioengineering (Basel). 2023 Mar 23;10(4):398. doi: 10.3390/bioengineering10040398 (PMC10136145; doi:10.3390/bioengineering10040398)
Supplement: Supplementary file 1 [file bioengineering-10-00398-s001.zip › bioengineering-2262290-supplementary.pdf]

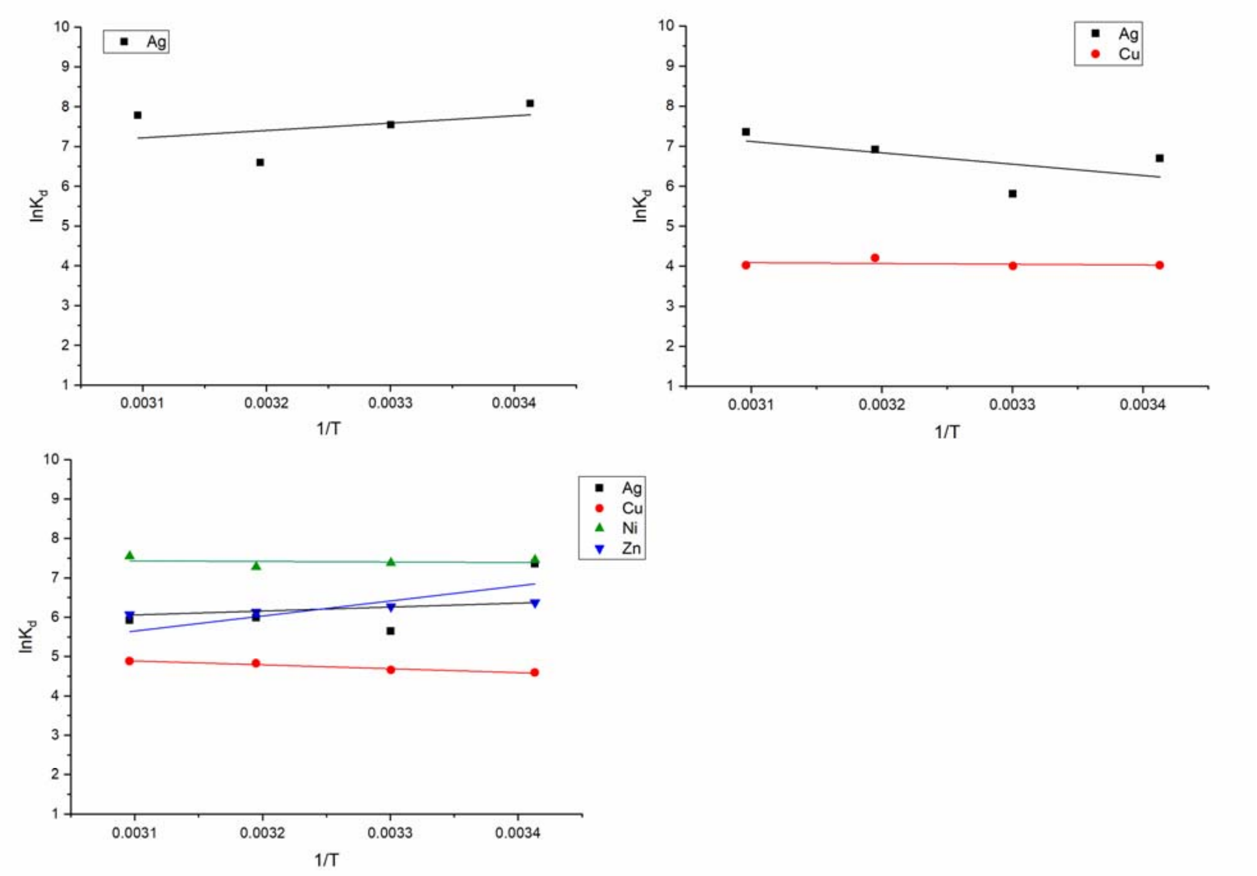

Figure S1: Plot of lnKd versus 1/T.

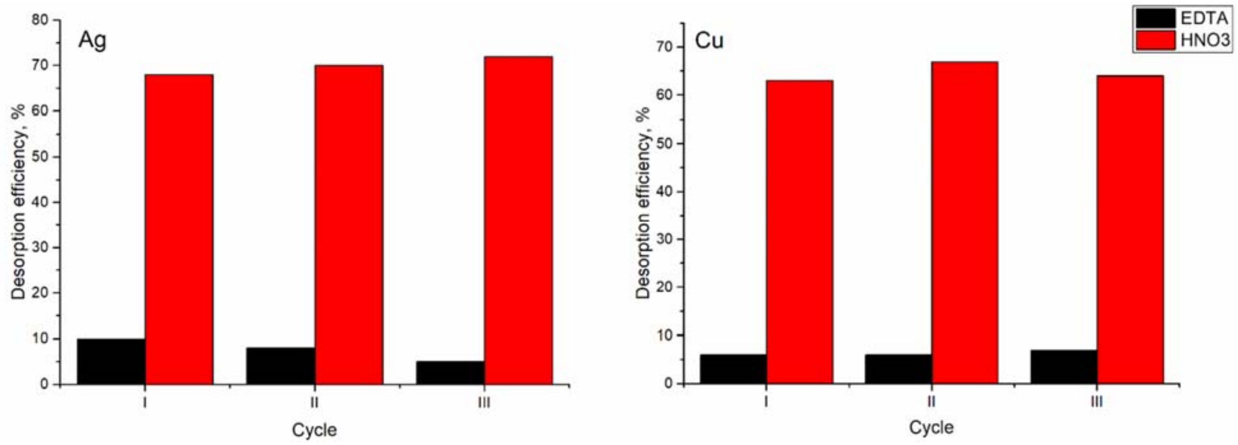

Figure S2: The efficiency of metal ions desorption using as eluents EDTA and HNO_3 in the Ag/Cu system.

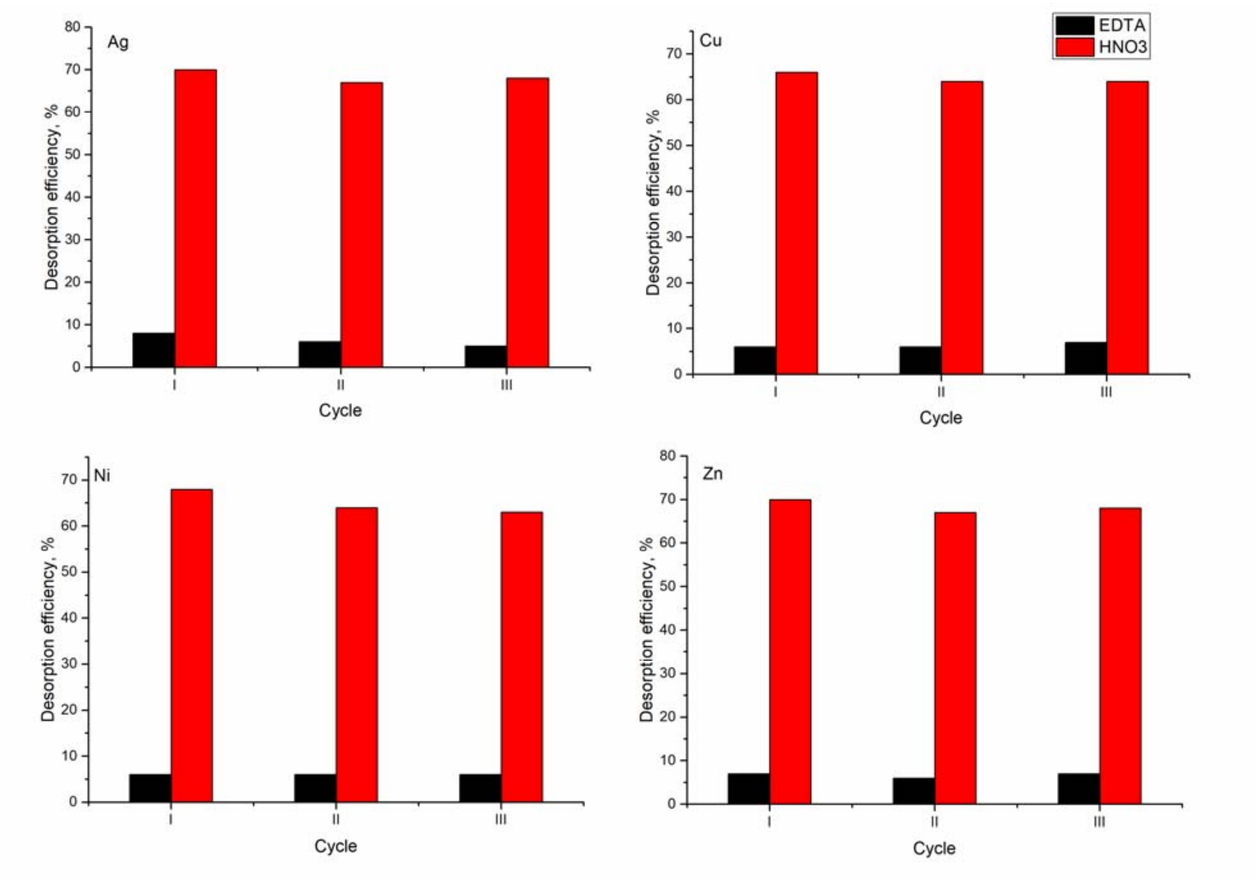

Figure S3: The efficiency of metal ion desorption using as eluents EDTA and HNO_3 in the Ag/Cu/Ni/Zn system.
